# Effects of Multiple Exposures and Ad-Skipping Behavior on Recall of Health Messages on YouTube^TM^

**DOI:** 10.3390/ijerph17228427

**Published:** 2020-11-14

**Authors:** Alexa R. Romberg, Shreya Tulsiani, Jennifer M. Kreslake, Erin J. Miller Lo, Bethany Simard, Amy Rask, Shruthi V. Arismendez, Donna M. Vallone, Elizabeth C. Hair

**Affiliations:** 1 Schroeder Institute, Truth Initiative, Washington, DC 20001, USA; alexaromberg@gmail.com (A.R.R.); stulsiani@truthinitiative.org (S.T.); jkreslake@truthinitiative.org (J.M.K.); erinjmlo@gmail.com (E.J.M.L.); bsimard@truthinitiative.org (B.S.); dvallone@truthinitiative.org (D.M.V.); 2 College of Global Public Health, New York University, New York, NY 10012, USA; 3 MediaScience, Austin, TX 78753, USA; a.rask@mediasciencelabs.com (A.R.); shruthi.vale@gmail.com (S.V.A.); 4 Department of Health, Behavior and Society, Bloomberg School of Public Health, Johns Hopkins University, Baltimore, MD 21205, USA

**Keywords:** outcome evaluation, adolescents, social marketing, health communications, tobacco control and policy

## Abstract

Although measuring exposure to public health messages is key to understanding campaign effectiveness, little is known about how exposure to and avoidance of digital ad messages may influence self-reported ad recall. A sample of 15–24-year-olds (*n* = 297) received a varying number of forced-view and skippable test ads across multiple simulated YouTube^TM^ sessions. Each session was coded for whether the participant viewed the ad or skipped it. While a majority of participants recalled the test ad, the odds of ad recall did not vary by number of sessions (opportunities for exposure). Rather, ad recall was sensitive to the number of completed ad views such that odds of ad recall doubled for each additional time the ad was completely viewed. Findings suggest that public health digital message exposure and recall can be optimized with sufficient attention paid to the proportions of forced-view ads aired when aiming to reach younger audiences.

## 1. Introduction

Evaluations of public health mass media campaigns are based on assessing the extent to which message exposure can influence attitudinal and behavioral outcomes. Such evidence requires careful measurement of message exposure and characterizing the sensitivity of exposure measures to true variations in exposure. Target Rating Points (TRPs) have long been used as a standard, objective exposure metric to quantify the reach and frequency of television advertising. However, the media landscape has changed dramatically over the past two decades: adolescents and young adults have shifted to viewing media primarily online, including through social media sites and streaming platforms. Today, health-related mass media campaigns are airing messages using digital platforms to help ensure sufficient exposure among youth and young adult target audiences [1,2,3]. The challenge for evaluators is that a standard, objective exposure metric, similar to TRPs for television advertising, is not currently available. 

In the absence of objective exposure measures, mass media campaign evaluators often rely on self-reported ad recall measures in which respondents are asked if they have seen ad messages either with or without a visual or text-aided prompt. Self-reported recall measures are widely used to determine brand and ad awareness in both commercial marketing and the scientific study of mass communication [4,5]. Validity of these measures has been supported by population-level correlations between self-reported and objective, exogenous measures such as television TRPs [6,7], and, more recently, individual-level associations with digital impressions [8]. 

Associations between self-reported ad recall and exogenous measures, however, are unable to capture the dynamics of how real-world exposure to ads leads to ad recall [9,10]. Understanding these dynamics in the digital space is important given that digital platforms are the primary vehicle through which media consumption occurs among youth and young adults [11]. Evidence suggests that digital ads are experienced differently than traditional television advertising. For example, digital ads are more likely to be viewed alone, and the active navigation within digital platforms creates a more interactive environment for the viewer than with television [12]. Moreover, advertising on digital platforms is viewed more negatively and as more intrusive than television advertising [13,14] and viewers tend to attempt to avoid internet ads, including learning to skip past ads when possible [9,15]. Ad avoidance reduces the level of engaged exposure that supports ad recall and subsequent effects on knowledge, attitudes, beliefs and behavior.

The purpose of this study was to better understand how digital ad recall is influenced by variations in ad viewing behavior. We examined self-reported ad recall within a simulated digital browsing context in which the test ad either played to completion or was skippable after the first five seconds of play. We tested two hypotheses: (1) that increasing *opportunities* for exposure would increase the odds of ad recall, and (2) that increasing the number of *completed ad views* (i.e., with no skipping) would increase odds of ad recall.

## 2. Methods

### 2.1. Sample 

Participants, aged 15–24, were recruited by a consumer neuroscience laboratory in Austin, Texas and Chicago, Illinois and randomly assigned to two (*N* = 101), three (*N* = 98), or four (*N* = 98) laboratory visits (total *N* = 297). An additional 37 participants were terminated from the study for failing to return for their next visit within 13 days and nine were excluded because of unusable data. Data were collected between August 2018 and April 2019. Informed consent and assent were provided by all participants and the guardians of minor participants. This study was approved by the Advarra IRB.

### 2.2. Study Procedures

Before beginning, participants were told that the study objective was to collect consumer evaluations of video content. The experimental web platform looked and functioned like a live YouTube site. YouTube was chosen as the digital platform for this study because of its popularity among youth and young adults [11,16]. Participants could navigate the trending videos page and select from 45 short-form videos, including sports, movie trailers, and others. The test ad was presented once during the 30-min YouTube session and played immediately before one of the selected videos. The test ad aired on television and digital platforms from February to August 2016 as part of the truth FinishIt anti-smoking campaign. To ensure that recall measured during the study was not attributable to potential prior exposure, a second group of participants (*N* = 281) was provided the same laboratory experience without the target ad. Ad recall in this control group was below 7% and so their data were not analyzed further for this report.

**Figure 1:**
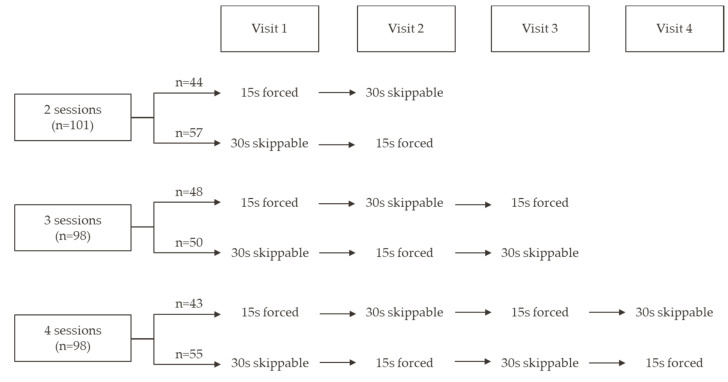
Digital exposure procedure.

Both 30-second and 15-second versions of the same test ad were used. Simulating the real-world presentation of ads on YouTube, the 15 s ad played all the way through before the selected video started (forced view) and the 30 s ad was skippable after the first five seconds of play. The 15 and 30 s versions of the test ad were alternated each week; half the participants were presented the 15 s ad in their first session and half the 30 s ad (Figure 1). 

During each lab session, participants were seated at a cubicle-style desk with privacy panels. YouTube videos were presented on a mounted Samsung Galaxy S7 mobile phone, allowing participants to interact with and adjust the device. Participants wore noise-cancelling headphones. Each session was video recorded.

Participants completed a survey after viewing the content of each session; only the survey during their final visit included questions about the test ad; other survey questions asked about the video content they had watched during the session. Participants were compensated each visit, with higher incentives on the last visit to encourage study completion.

### 2.3. Measures

The self-report outcome measure was similar to those used in recent evaluations of national anti-smoking campaigns [17,18]. Participants were shown a collage of screenshots from the test ad with the text, “Below are screenshots from an ad from truth that you may have been shown during your experiences with us. Please review these images and answer the following questions.” Participants were then asked, “Did you watch this ad in your session today?” with response options, “Yes” or “No.” 

### 2.4. Analysis Plan

The proportion of respondents who recalled the ad was calculated for each number of sessions. A logistic regression model was used to test the effect of number of sessions (opportunities for exposure) on ad recall. 

While a participant may have attended four sessions and was presented with the ad four times, they may have opted to skip the 30 s ad after five seconds of play and reduced their actual amount of exposure. We conducted a second analysis that grouped participants based on their completed ad views, as opposed to opportunities to view. The number of completed ad views was calculated for each participant as the sum of number of forced-view 15 s ads shown and the number of 30 s ads the participant let play for at least 15 s. Logistic regression was used to estimate the likelihood of self-reported ad recall with respect to the number of completed views.

Because participants were not randomly assigned to the number of completed views, covariates were included in all regression models to control for any potential differences in ad recall by demographic groups. All patterns of significance are the same when the models are run on the full sample without covariates. All confidence intervals reported are at the 95% level.

## 3. Results

### 3.1. Sample Characteristics

Sample demographics are summarized in Table 1. The sample was evenly split by gender with a mean age of 20 years. Most participants had never used cigarettes, and approximately 11% were current (past 30 days) smokers.

### 3.2. Ad Recall by Number of Sessions

Self-reported ad recall was 80.2% (CI: 70.8–87.2%) for those who came in for two sessions, 70.4% (CI: 60.2–79.0%) for those who came in for three sessions, and 81.6% (CI: 72.3–88.5%) for those who came in for four sessions. 

Logistic model results are shown in Table 2. There was not a significant relationship between number of sessions and odds of ad recall (OR = 1.09, CI: 0.75–1.59). The only covariate that significantly predicted ad recall was participant race, with Hispanic (OR = 0.30, CI: 0.14–0.65) and non-Hispanic black (OR = 0.15, CI: 0.06–0.39) participants both having lower odds of ad recall compared to non-Hispanic white participants. Cigarette use was not significantly associated with ad recall.

### 3.3. Ad Skipping and Recall

Participants skipped most of the 30 s skippable ads and skipping increased slightly across the sessions: 73.5%, 74.0%, 82.9%, and 83.3% of 30 s ads were skipped within the first 15 s in Sessions 1, 2, 3, and 4, respectively, across all participants. 

Further analyses were conducted to determine whether the counterbalancing component of the study design, which alternated the forced-view and the skippable ads, contributed to the lower ad recall among participants who attended for three rather than two sessions. Of the three-session participants, half were presented with two forced-view ads and one skippable ad, and half were presented with two skippable ads and one forced-view ad. Participants who were presented with two forced-view ads had higher ad recall (76.0%, CI: 63.7–88.3%) than those who were presented with two skippable ads (64.6%, CI: 50.5–78.6%), but this difference did not reach statistical significance (Pearson χ^2^ = 1.53, *p* = 0.22).

When participants were grouped by the number of completed views across all sessions, the ad recall rate was 72.2% (CI: 62.9–79.9%) for those with one completed view (*N* = 109), 76.9% (CI: 68.6–83.5%) for those with two completed views (*N* = 134), 92.1% (CI: 77.5–97.9%) for those with three complete views (*N* = 43), and 88.9% (CI: 50.7–99.4%) for those with four completed views (*N* = 11).

Table 2 presents results from the logistic regression model predicting ad recall by the number of complete ad views. Each additional complete view doubled the odds of ad recall (OR = 2.00, CI: 1.27–3.13). There was also a significant relationship between participant race/ethnicity and ad recall, with Hispanic (OR = 0.25, CI: 0.11–0.56) and non-Hispanic black (OR = 0.15, CI: 0.06–0.39) participants both having lower odds of ad recall that non-Hispanic white participants. No other covariates, including cigarette use, were associated with ad recall.

## 4. Discussion

Accurately quantifying exposure to campaign messaging is a critical component in evaluating mass media public health campaigns. This study provides evidence that exposure to ad messages on YouTube prompts self-reported ad recall, and that message recall is directly related to completed ad viewing. Overall, over three-quarters of the participants recalled the ad, and each completed ad view doubled the odds of recall. 

This is the first study that we know of to examine the impact of skipping digital ads on ad recall of a public health message. We found that the likelihood of ad recall did not increase with opportunities for exposure when participants were able to sometimes skip the ad because most participants skipped ads when possible. Thus, skipped ads did not serve as an effective memory prompt for participants even though a small portion, five seconds, of the ad played before the skipping was allowed. It is possible that the design of the ad used contributed to the lack of effect for skipped ads. Neither the truth branding nor the primary message was displayed within the first five seconds of the ad. It is possible that ads designed specifically to maximize the impact of the first 5 s might produce a different result.

Recall increased with each completed ad view, and this effect was present even though completed ad views often occurred on non-consecutive weeks. This pattern validates that self-reported recall measures are sensitive to true variation in exposure. Prior work has found that those with personal experience with the subject matter of the campaign are more likely to recall the messaging [19,20]. However, we did not find an association between cigarette use and ad recall in this study. This lack of association could be due to the lab-based study design, which may have ameliorated effects of participant characteristics.

These findings extend those from a recent study which found that self-reported ad recall had a dose–response relationship with the number of times an ad had been encountered during regular web browsing [8]. Results suggest that a dose–response relationship between actual message exposure and message recall persists, even within a digital experience. Thus, self-report measures remain a useful tool for health communication researchers and can be used to address measurement challenges for campaign evaluation as the media landscape shifts from television to digital [10].

### Limitations and Strengths

While there are many strengths to this study, there are several limitations. The level of self-reported ad recall under study conditions may not reflect levels of ad recall in real-world viewing experiences. In particular, recall was measured very soon after the last exposure, but one expects recall to decay over time and would likely be lower if measured at a later point. Participants may have paid more attention than usual to the ads because of the lab environment. The level of ad recall may also have been enhanced since the ad had aired on television prior to the study. However, the low rate of ad recall in participants whose visits did not include the test ad suggests this effect would be small. Given the random assignment to number of sessions, these limitations should not affect the relative differences in ad recall with varying degrees of experimental exposure.

## 5. Conclusions

The changing media landscape presents both opportunities and challenges for evaluating public health campaigns. Recall of health messages is the first step in changing attitudes, beliefs, and behaviors. Findings from this study suggest that using digital media such as YouTube can prompt reliable ad recall that varies with the amount of true exposure. Importantly, audience recall of digital ads requires repeated exposure to the intended message, rather than simply a visual cue in the first few seconds of a skippable ad (e.g., a logo, tagline, or mascot) to prompt recall. Digital media platforms offer a cost-effective approach for public health campaigns to reach specific target audiences. The inclusion of forced-view ads in digital media planning is recommended to help ensure campaign effectiveness. 

## Figures and Tables

**Table 1:** Participant Characteristics.

Variable	Overall *N* (%)	Exposed *N* (%)	Control *N* (%)
**Mean age ± SD (years)**	20.0 ± 2.8	20.0 ± 2.9	20.0 ± 2.8
**Gender**			
Male	290 (50.3)	147 (49.8)	143 (50.9)
Female	286 (49.7)	148 (50.2)	138 (49.1)
**Race**			
Non-Hispanic White	241 (43.3)	122 (42.5)	119 (44.2)
Non-Hispanic Black	84 (15.1)	48 (16.7)	36 (13.4)
Hispanic	163 (29.3)	85 (29.6)	78 (29.0)
Non-Hispanic Other	68 (12.2)	32 (11.1)	36 (13.4)
**Financial Situation**			
Do not meet basic needs	46 (8.1)	25 (8.5)	21 (7.6)
Just meet basic needs with nothing left over	168 (29.5)	86 (29.4)	82 (29.6)
Meet basic needs with little left over	222 (38.9)	113 (38.6)	109 (39.4)
Live comfortably	134 (23.5)	69 (23.5)	65 (23.5)
**Cigarette Use**			
Never	360 (63.4)	182 (62.5)	178 (64.3)
Ever but not current	147 (25.9)	79 (27.1)	68 (24.5)
Current	61 (10.7)	30 (10.3)	31 (11.2)

*Note*. Due to missing data, sample sizes for each measure may not have the same sums.

**Table 2:** Effects of Exposure by Session or by Complete Views on Self-Reported Ad Recall (*n* = 278).

Variable	OR (95% CI)	OR (95% CI)
**Exposure**		
Number of Sessions ^a^	1.09 (0.75, 1.59)	-
Number of Completed Views ^b^	-	2.00 (1.27, 3.13) **
**Age**	1.05 (0.93, 1.19)	1.03 (0.91, 1.17)
**Gender**		
Female	REF	REF
Male	1.21 (0.64, 2.31)	1.30 (0.67, 2.52)
**Race/Ethnicity**		
NH White	REF	REF
NH Black	0.15 (0.06, 0.39) ***	0.15 (0.06, 0.39) ***
Hispanic	0.30 (0.14, 0.65) **	0.25 (0.11, 0.56) ***
NH Other	0.71 (0.23, 2.22)	0.73 (0.23, 2.33)
**Financial Situation**		
Do not meet basic needs	REF	REF
Just meet basic needs with nothing left over	2.17 (0.65, 7.23)	2.49 (0.74, 8.39)
Meet basic needs with little left over	0.85 (0.29, 2.53)	1.17 (0.38, 3.58)
Live comfortably	0.46 (0.14, 1.52)	0.61 (0.18, 2.01)
**Cigarette Use**		
Never	REF	REF
Ever but not current	0.58 (0.27, 1.23)	0.64 (0.30, 1.38)
Current	0.34 (0.12, 1.01)	0.35 (0.12, 1.04)

OR = odds ratio; CI = confidence interval. ** *p* < 0.01; *** *p* < 0.001. ^a^ Session (2, 3, or 4) is the number of laboratory visits to which the participant was randomly assigned, with the test ad presented once per session. ^b^ Completed ad views (1, 2, 3, or 4) is the number of test ads that the participant viewed (i.e., did not skip) across all sessions attended.
